# Reduced fire blight susceptibility in apple cultivars using a high‐efficiency CRISPR/Cas9‐FLP/FRT‐based gene editing system

**DOI:** 10.1111/pbi.13253

**Published:** 2019-10-03

**Authors:** Valerio Pompili, Lorenza Dalla Costa, Stefano Piazza, Massimo Pindo, Mickael Malnoy

**Affiliations:** ^1^ Department of Genomics and Biology of Fruit Crops Research and Innovation Centre Fondazione Edmund Mach San Michele all'Adige Italy; ^2^ Department of Agricultural, Food, Environmental and Animal Sciences Università degli Studi di Udine Udine Italy

**Keywords:** *Malus* × *domestica*, fire blight, DIPM, gene editing, FLP/FRT recombination

## Abstract

The bacterium *Erwinia amylovora*, the causal agent of fire blight disease in apple, triggers its infection through the DspA/E effector which interacts with the apple susceptibility protein MdDIPM4. In this work, *MdDIPM4* knockout has been produced in two *Malus* × *domestica* susceptible cultivars using the CRISPR/Cas9 system delivered *via Agrobacterium tumefaciens*. Fifty‐seven transgenic lines were screened to identify CRISPR/Cas9‐induced mutations. An editing efficiency of 75% was obtained. Seven edited lines with a loss‐of‐function mutation were inoculated with the pathogen. Highly significant reduction in susceptibility was observed compared to control plants. Sequencing of five potential off‐target sites revealed no mutation event. Moreover, our construct contained a heat‐shock inducible FLP/FRT recombination system designed specifically to remove the T‐DNA harbouring the expression cassettes for CRISPR/Cas9, the marker gene and the FLP itself. Six plant lines with reduced susceptibility to the pathogen were heat‐treated and screened by real‐time PCR to quantify the exogenous DNA elimination. The T‐DNA removal was further validated by sequencing in one plant line. To our knowledge, this work demonstrates for the first time the development and application of a CRISPR/Cas9‐FLP/FRT gene editing system for the production of edited apple plants carrying a minimal trace of exogenous DNA.

## Introduction

Apple (*Malus* × *domestica*) is one of the most cultivated fruit crops throughout the temperate regions of the world. Its production faces continual new challenges such as a constant change in consumer demand, based on a variation of tastes and flavours, and, from an agronomic point of view, climate change and harmful biotic agents (insects or bacterial, fungal and viral pathogens). Among them, one of the major economic threats to apple production worldwide is the necrogenic and highly infectious Gram‐negative bacterium *Erwinia amylovora* (Burrill; Winslow *et al*., 1920), the causative agent of fire blight disease.

*Erwinia amylovora* uses a complex regulatory network of many virulence determinants to establish infection. However, recent studies have been particularly focused on DspA/E, a 198 kDa effector protein homologous to the type III effector AvrE of *Pseudomonas syringae* pv. *tomato* (Gaudriault *et al*., 1997). It has been shown that the capacity of the bacterium to induce disease mainly depends on this single delivered effector (Siamer *et al*., 2014). Degrave *et al*. (2013) showed that DspA/E was required for transient bacterial growth in nonhost *Arabidopsis thaliana* leaves, while an *E. amylovora dspA/E* mutant was unable to grow. In addition, after its secretion into the cytoplasm of host plant cells, DspA/E was shown to interact with the intracellular domains of host plant receptor kinases (Boureau *et al*., 2006; Oh *et al*., 2007). In apple, using a yeast two‐hybrid assay and an *in vitro* protein pull‐down assay, Meng *et al*. (2006) demonstrated that DspA/E physically and specifically interacts with the kinase domain of four leucine‐rich repeat (LRR) receptor‐like serine/threonine kinases (RLK), called DspA/E‐interacting proteins of *M. × domestica* (DIPM1 to DIPM4). The structures of DIPMs indicate that they might function in signal transduction, perhaps by sensing extracellular signals with LRRs and interacting with effectors through the RLKs. Thus, Meng and colleagues suggested that the interaction of DspA/E with the DIPMs may suppress a defence response by interrupting DIPM signal transduction and that DIPM proteins may act as susceptibility factors during the *E. amylovora*–apple interaction.

Among the most advanced technologies of genetic engineering (New Breeding Technologies, NBT), gene editing *via* the clustered regularly interspaced short palindromic repeats (CRISPR)/CRISPR‐associated protein 9 (CRISPR/Cas9) has emerged as an effective tool for gene functional analysis in plants. It can directly introduce mutations into the plant genome by operating through guide RNA (designed to target a specific genomic sequence) and the Cas9 protein (which cleaves the specific site within the target gene) activating the error‐prone non‐homologous end‐joining (NHEJ) pathway for DNA repair (Barabaschi *et al*., 2016). CRISPR/Cas9 system, which involves simple designing and cloning methods, has emerged as a powerful strategy to precisely and quickly insert the desired traits into a plant genome, with the aim of facing biotic and abiotic stresses as well as improving other important agronomic traits (Jaganathan *et al*., 2018). To date, there have been many studies reporting the use of CRISPR/Cas9 in plants of agricultural interest, such as tomato (Pan *et al*., 2016; Ueta *et al*., 2017), potato (Andersson *et al*., 2017), wheat (Gil‐Humanes *et al*., 2017; Liang *et al*., 2017), orange (Jia and Wang, 2014), grape (Malnoy *et al*., 2016; Nakajima *et al*., 2017; Ren *et al*., 2016; Wang *et al*., 2018), pear (Charrier *et al*., 2019) and apple (Charrier *et al*., 2019; Malnoy *et al*., 2016; Nishitani *et al*., 2016). In plants, one of the most recent applications of CRISPR/Cas9 relies on the delivery of the Cas9‐guide RNA ribonucleoprotein complex directly into plant protoplasts. Following the generation of mutations at the targeted genomic site, the Cas9‐guide RNA complex is processed and degraded resulting in edited protoplasts free from exogenous editing machinery, some of which can be regenerated into a new plant. In *Arabidopsis*, lettuce, petunia, rice, tobacco and wheat, this methodology was successfully reported (Subburaj *et al*., 2016; Woo *et al*., 2015; Zhang *et al*., 2018). Nevertheless, the strategy can neither be widely applied nor represent an alternative to the conventional *Agrobacterium tumefaciens* (*A. tumefaciens*)‐mediated transformation as, to date, efficient protocols for protoplast regeneration are still not available for many plant species. In fact, especially in apple, applications of the CRISPR/Cas9 system still rely on conventional transformation methodologies *via A. tumefaciens*, which, however, lead to the production of edited plants containing exogenous DNA.

Multiple recombination systems such as Cre/loxP, R/Rs or FLP/FRT have been developed to remove unwanted foreign DNA elements from transformed crops with the aim of alleviating consumer and regulatory concerns. These approaches for transgene elimination are based on transformation vectors containing the recombinase gene and transgenes between two directly repeated recombinase recognition sites (RRS). For many crops, these recombination systems have been successfully used to excise selectable marker genes from the genome of transgenic plants. For instance, in transgenic apple (Herzog *et al*., 2012; Righetti *et al*., 2014; Würdig *et al*., 2013) and pear (Righetti *et al*., 2014), proofs of concept were conducted to investigate the feasibility of eliminating the *nptII* marker gene using the FLP/FRT and R/Rs recombination systems. Similarly, Dalla Costa *et al*. (2016) demonstrated the removal of *nptII* gene in grape. Moreover, from an applicative point of view, these recombination systems were also applied in transformation vectors to produce cisgenic plants carrying the genetic trait of interest and free from undesirable T‐DNA sequences. In the work of Kost *et al*. (2015), to improve fire blight resistance in a susceptible apple cultivar, one plant was generated using the *FB_MR5* gene in a cisgenic approach based on the FLP/FRT recombinase system. In addition, scab resistance was improved in different apple cultivars by a similar cisgenic approach to introduce the *Rvi6* gene into the apple genome (Würdig *et al*., 2015).

In this work, we have used a CRISPR/Cas9 gene editing approach to knockout the *MdDIPM4* susceptibility gene to reduce fire blight susceptibility in *M. × domestica* cultivars, ‘Gala’ and ‘Golden Delicious’. Moreover, a strategy based on the heat‐shock inducible FLP/FRT recombination system was applied to remove the T‐DNA region containing expression cassettes of the editing machinery and selectable marker in those edited lines with reduced susceptibility to the disease.

## Results

### Generation of edited transgenic apple lines

**Figure 1:**
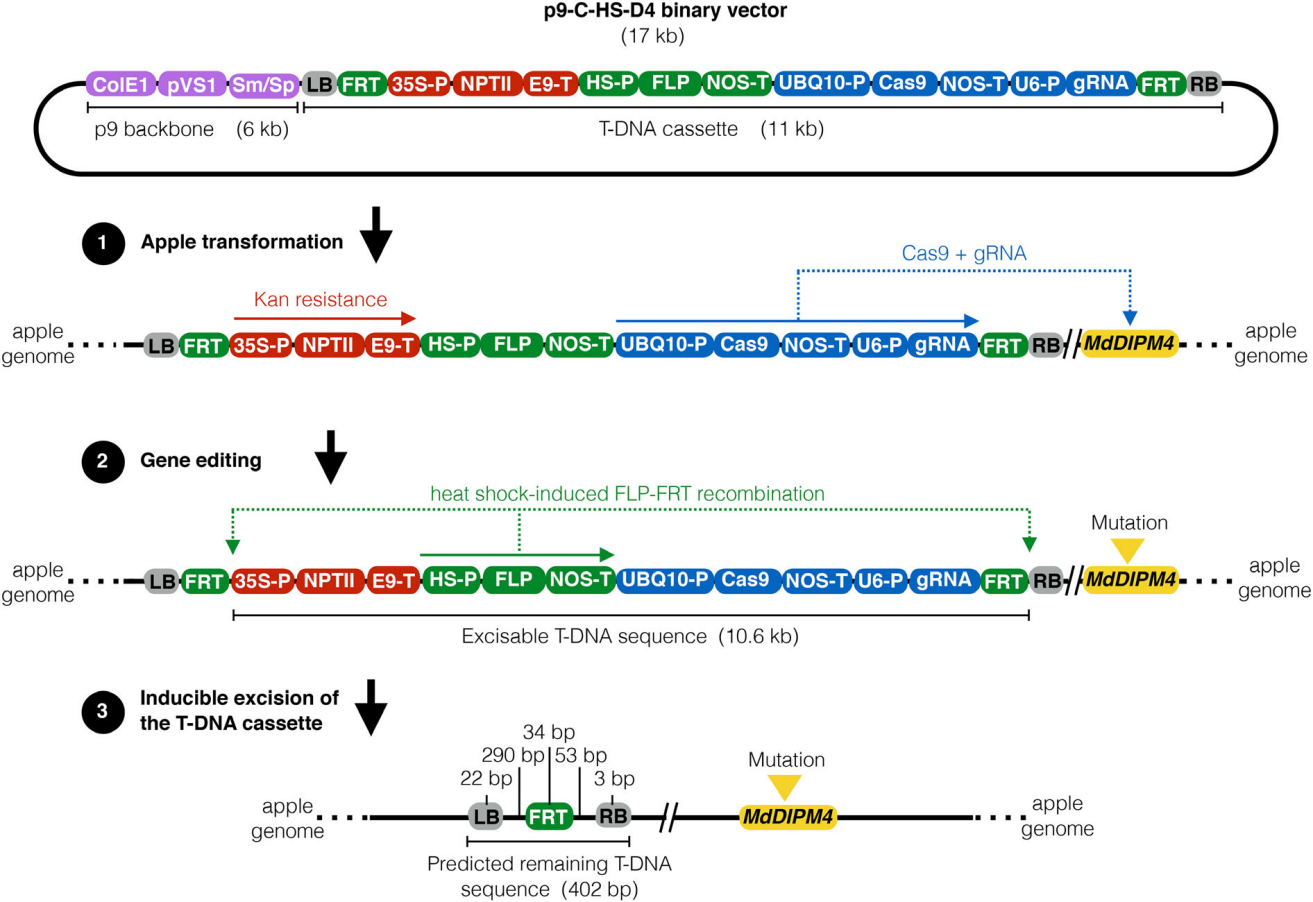
Schematic representation of structure and mechanism of action of the p9‐C‐HS‐D4 binary vector based on the CRISPR/Cas9‐FLP/FRT gene editing system. The binary vector is produced by cloning a 11 kb T‐DNA cassette into a 6 kb p9 vector backbone (violet boxes). The T‐DNA cassette contains a kanamycin resistance system (red boxes) for plant selection after the apple transformation (step 1), a gene editing system (blue boxes) to target genomic *MdDIPM4* (yellow box) (step 2) and a heat‐shock inducible FLP/FRT recombination system (green boxes) for the excision of the exogenous DNA (step 3). *Colicin E1* and *pVS1* origins of replication (ColE1 and pVS1); streptomycin/spectinomycin resistance genes (Sm/Sp); left and right borders (LB and RB); flippase recognition target site (FRT); *Cauliflower Mosaic Virus 35S* promoter (35S‐P); *Neomycin phosphotransferase II* (NptII); E9 terminator (E9‐T); heat‐shock inducible promoter (HSP); *Flippase* gene (FLP); *Nopaline Synthase* terminator (NOS‐T); *Arabidopsis thaliana Ubiquitin‐10* promoter (UBQ10‐P); *Crispr‐associated protein 9* (Cas9); *Arabidopsis thaliana U6* promoter (U6‐P); guide RNA for *MdDIPM4* target (gRNA); and kanamycin (Kan).

A total of 2000 ‘Gala’ and 1370 ‘Golden Delicious’ leaf explants were infected with *A. tumefaciens* containing the binary vector carrying the CRISPR/Cas9 machinery (Figure 1), specifically targeting a *MdDIPM4* region with no homology to other members of the DIPM gene family (Figure S1). The target site is identical between the two cultivars without allelic variations. Respectively, 40 and 46 regenerants of ‘Gala’ and ‘Golden Delicious’ were collected approximately 6–7 months after culture in selective medium (Table 1). Regenerants were tested by PCR to screen for the integration of T‐DNA (primer listed in Table S1). A total of 31 ‘Gala’ and 35 ‘Golden Delicious’ apple lines had the *Cas9* gene integrated in the genome and no *A. tumefaciens* contamination, resulting in transformation efficiencies of 1.55% and 2.55%, respectively (Table 1).

**Table 1:** Efficiency of *Agrobacterium tumefaciens*‐mediated transformations in ‘Gala’ and ‘Golden Delicious’ apple cultivars

Cultivars	Transformation name	No. of leaf explants infected	No. of regenerants collected	PCR screening			Transformation efficiency (%)†
				No. of regenerants tested	No. of positive regenerants		
					*Cas9*	*VirG*	
Gala	V1	920	29	27	24	0	1.55
	V3	1080	11	8	8	1	
Golden Delicious	V2	330	6	3	3	0	2.55
	V4	1040	38	32	32	0	

^†^ The transformation efficiency was calculated by dividing the number of regenerants positive for *Cas9* and negative for *VirG* by the number of leaf explants infected (considering V1 + V3 and V2 + V4).

### Characterization of *MdDIPM4* mutants and selection of candidate apple lines

**Figure 2:**
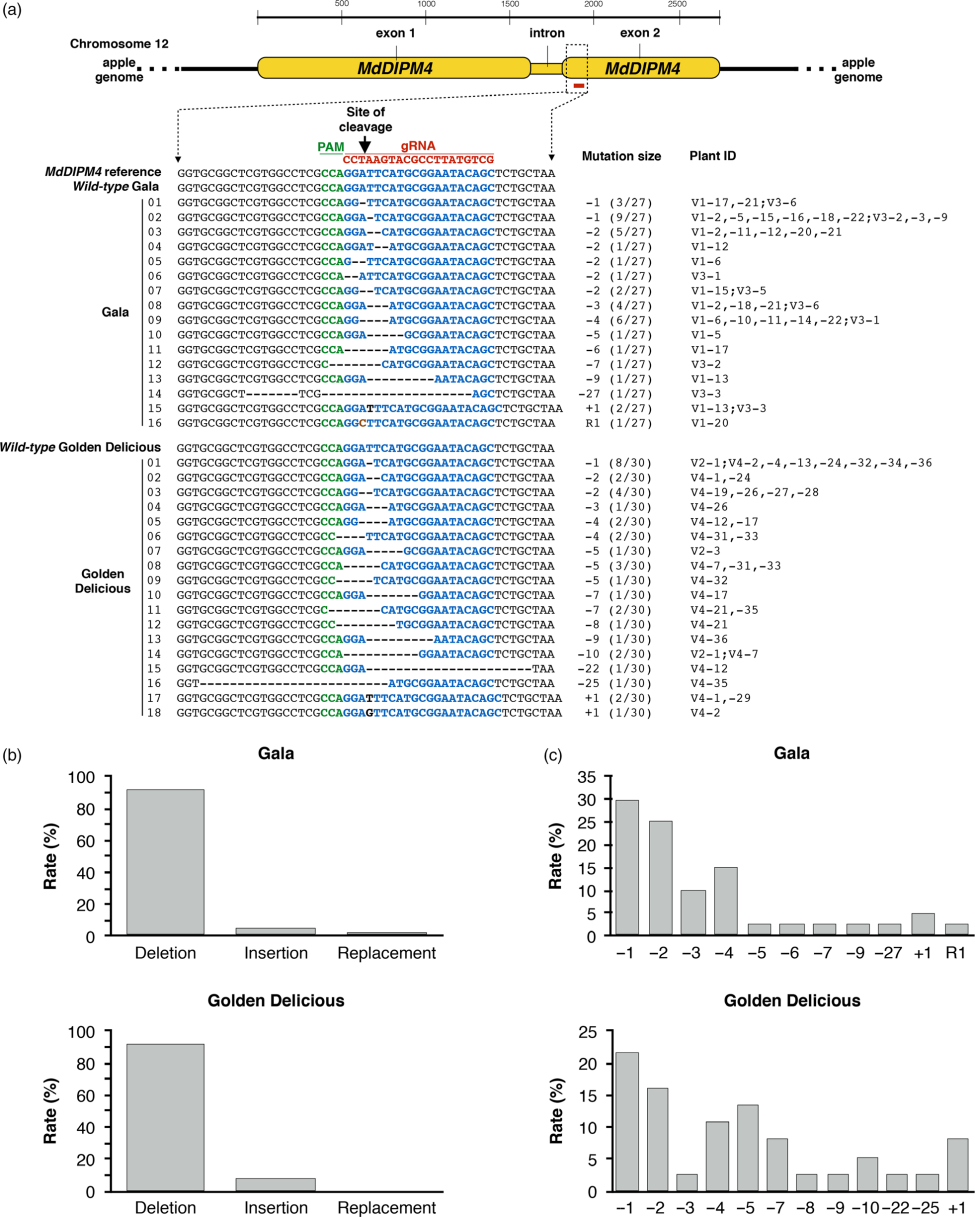
CRISPR/Cas9‐editing in *Malus* × *domestica* cultivars ‘Gala’ and ‘Golden Delicious’ detected by HTS. (a) Representation of NHEJ mutation events generated by the CRISPR/Cas9 system in *MdDIPM4* gene. NHEJ mutations were detected on the predicted site of cleavage using as reference the *MdDIPM4* genomic sequence of the apple genome assembly GDDH13 v1.1 (Daccord *et al*., 2017) in addition to *wild‐type* plants. The *MdDIPM4* target sequence is coloured in blue. Within the sequence alignment, deletions are represented by traits. Insertions and replacement are shown, respectively, with black and orange bold letters. Mutations and plant ID are shown on the right. Numbers on the left refer to those reported in Figure 3. Guide RNA (gRNA); protospacer adjacent motif (PAM). (b) Rate of mutation types. (c) Rate of mutation sizes. Percentages in (b) and (c) were calculated by dividing the number of total events (respectively, of each mutation type and size) by the sum of total mutation events.

The *MdDIPM4* target region was screened in 57 transgenic lines (respectively, 27 and 30 for ‘Gala’ and ‘Golden Delicious’ backgrounds, hereafter ‘G’ and ‘GD’) using high‐throughput sequencing (HTS) on the Illumina MiSeq Platform (Figure 2a). On average, 3000 raw sequence reads were obtained for each of the analysed plants. Editing results were very similar between the two cultivars, showing a percentage of non‐edited plants of 22.2% for ‘G’ and 26.7% for ‘GD’, corresponding to editing efficiencies of 77.8% and 73.3%, respectively. Among the edited plants, some were completely edited showing a single type (homozygous) mutation (7.4% ‘G’—20% ‘GD’) or multiple mutation (heterozygous) profiles (63% ‘G’—50% ‘GD’), while others had a partially edited genotype as a *wild‐type* background was maintained (7.4% ‘G’ and 3.3% ‘GD’). Several types of mutation were identified as follows: a small insertion (+1 nt), a small replacement (R1 nt), small deletions (−1, −2, −3, −4, −5, −6, −7, −8, −9, −10 nts) and large deletions (−22, −25, −27 nts; Figure 2a). The most frequent type of mutation was nucleotide deletion, with a 91.7% in ‘GD’ and a 92.5% in ‘G’, followed by nucleotide insertion (5% ‘G’ and 8.3% ‘GD’) and nucleotide replacement (2.5% only for Gala; Figure 2b). In both apple cultivars, −1 nt (21.6% ‘GD’—30% ‘G’) and −2 nts (16.2% ‘GD’—25% ‘G’) were the most abundant, followed by −3 nts (10%) and −4 nts (15%) for ‘G’ and −4 nts (10.8%) and −5 nts (13.5%) for ‘GD’ (Figure 2c).

These data were partially consistent with a previous analysis based on Sanger sequencing (Sanger experimental procedure in Supporting Information) of the target site (Figure S2) according to which three plant lines (V1‐4, V1‐7 and V4‐3) did not contain mutations and the others showed a short insertion (+1 nt) and short deletions (−1, −2, −3, −4, −5, −6, −10 nts). The discrepancy in the results produced by the two sequencing methods concerned the profile of two plant lines (V1‐6 and V4‐7).

**Figure 3:**
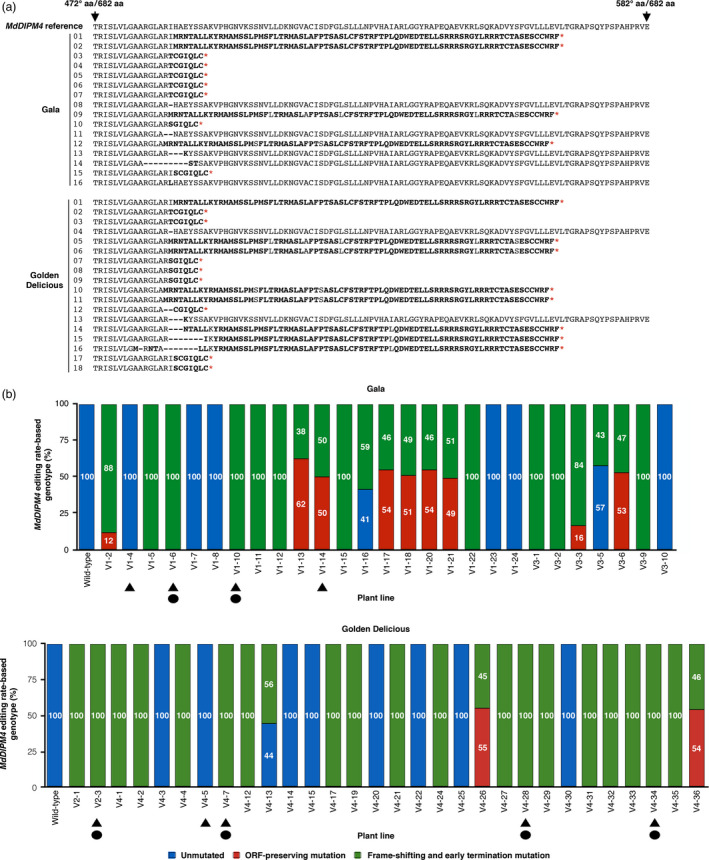
MdDIPM4 protein and *MdDIPM4* editing rate‐based genotypes of CRISPR/Cas9‐edited apple plants. (a) A fragment of the translated MdDIPM4, from 472 to 582 out of 682 amino acids (aa), containing the CRISPR/Cas9 site of cleavage is shown. Amino acids in bold indicate transcoding sequences compared to the MdDIPM4 reference. Deleted amino acids and stop codons are represented, respectively, by traits and red asterisks. Numbers on the left refer to those reported in Figure 2. (b) The summary of *MdDIPM4* editing rate‐based genotypes of transgenic apple plants compared to *wild‐type* plants is reported. Triangles and circles indicate candidate apple lines selected, respectively, for the analysis of plant resistance to *Erwinia amylovora* and heat‐shock inductions.

The deduced amino acid sequences of the MdDIPM4 protein for all the analysed plants are shown in Figure 3a. A total of 16 and 18 MdDIPM4 edited sequences were transduced for ‘G’ and ‘GD’, respectively. In ‘G’, 11 sequences showed frame‐shifting and early termination mutations with the introduction of stop codons responsible for the premature termination of protein transduction. The remaining five sequences showed ORF‐preserving mutations that caused the loss of one or few amino acids without affecting the protein translation. In ‘GD’, 16 sequences had early termination mutations and only 2 ORF‐preserving mutations (Figure 3a). The summary of the editing rate of *MdDIPM4* for all the transgenic apple plants confirmed with T‐DNA insertion for both cultivars is shown in Figure 3b.

All the transgenic plants evaluated for the editing in the target site were subsequently characterized for T‐DNA integration copy number (CN) by quantifying the *nptII* selection marker gene (Table S2). *NptII* CN ranged from a minimum of 0.2 to a maximum of 4.9. According to mutation profiles and T‐DNA integration CN, lines V1‐4, V1‐6, V1‐10 and V1‐14 for ‘G’ and V2‐3, V4‐5, V4‐7, V4‐28 and V4‐34 for ‘GD’ (Figure 3b) were used for the subsequent assays of fire blight resistance and removal of the T‐DNA following the workflow shown in Figure S3 (workflow experimental procedure in Supporting Information). Those lines were re‐screened by HTS in order to confirm their editing profile after 6 months of micropropagation (corresponding to 7 months after the regeneration of transgenic plants). The analysis of the reads obtained (ca. 10 000 raw reads/plant) confirmed the previous data (data not shown). Moreover, with regard to putative pleiotropic effects caused by the knockout of *MdDIPM4*, selected plant lines were observed during several rounds of micropropagation and acclimation to soil and no visible phenotypic differences were found between *wild‐type* and transgenic plants.

### Fire blight resistance test in *MdDIPM4* knockout mutants

**Figure 4:**
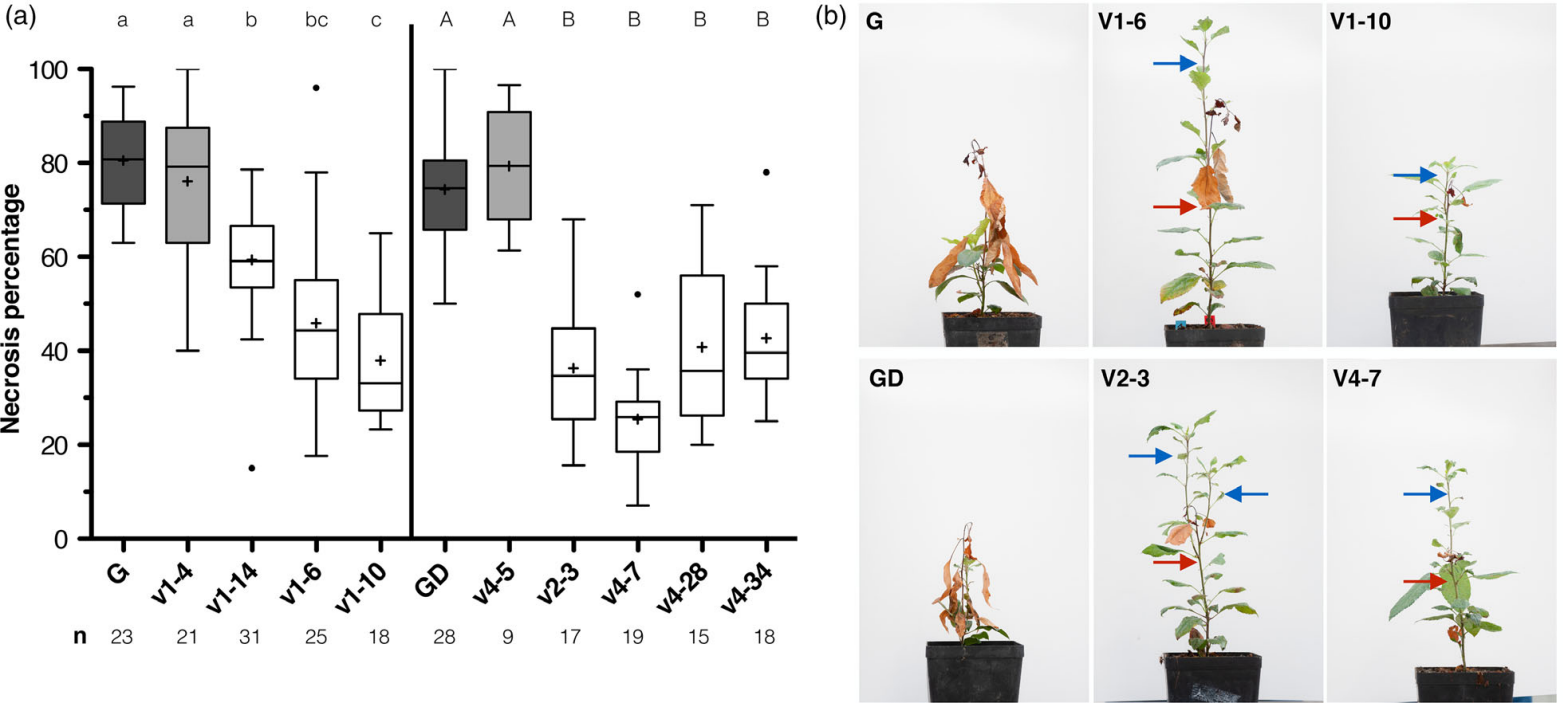
Fire blight severity in *MdDIPM4*‐edited plants of ‘Gala’ and ‘Golden Delicious’ cultivars. (a) Boxplot summarizing the percentage of necrosis (calculated as length of the necrosis/total length of the shoot × 100) of candidate *MdDIPM4* CRISPR/Cas9‐edited plants inoculated by the method of scissor with *Erwinia amylovora* strain Ea273. The number of inoculated biological replicates for each line is indicated (n). Boxes comprise values between 25% and 75% of the group. Horizontal central lines represent medians. Mean is shown as + . Whiskers (Tukey) determine values within ±1.5 interquartile ranges from the median. Circles indicate outliers. Lettering indicates statistically significant differences between plant lines (for ‘Gala’ lower case, for ‘Golden Delicious’ upper case) according to Kruskal–Wallis test followed by multiple comparison of mean rank (α = 0.05). (b) Pictures, taken 1 month after inoculation, showing the fire blight‐induced necrotic phenotype in *wild‐type* and some transgenic lines. Red and blue arrows indicate the interruption of necrosis and new regenerated shoots, respectively. Gala (G); Golden Delicious (GD).

Selected apple lines were infected with *E. amylovora* strain Ea273 in three independent experiments. Results of necrotic symptoms are shown in Figure 4. For the cultivar ‘G’, *wild‐type* plants (control) were susceptible showing a necrosis percentage of 81% ± 10% (Figure 4a). Similarly, one transgenic but non‐edited line (V1‐4) had a necrosis phenotype of 76% ± 16%. On the contrary, all other edited lines showed a highly significant (*P*‐value < 0.001) reduction in symptoms compared to control. The percentage of necrotic symptoms varied from 61% ± 10% for the chimeric line V1‐14 to 44% ± 18% for the line V1‐6 and 35% ± 11% for the line V1‐10. For the cultivar ‘GD’, control plants and the transgenic but non‐edited line V4‐5 had similar necrosis percentages of 73% ± 9% and 79% ± 13%, respectively. Conversely, all other edited lines showed a highly significant (*P*‐value < 0.001) reduction in susceptibility compared to control. Lines V2‐3, V4‐28 and V4‐34 had a necrosis percentage of, respectively, 35% ± 12%, 40% ± 15% and 42% ± 11%, while for the line V4‐7 the disease symptoms were even lower showing a necrosis percentage of 25% ± 11% (Figure 4a). In summary, for ‘G’ and ‘GD’ completely edited lines symptoms were reduced on average of 50%. The ‘G’ line V1‐14 showed a lower reduction (25%) due to its chimeric profile. For this line, the presence of T‐DNA chimeric tissues was based on the *nptII* CN lower than 1 (i.e. = 0.6). In this case, the partial editing rate in the target site (half edited/half *wild‐type;* Figure 3b) is due to chimerism and not to a heterozygous state. Moreover, in both cultivars, at the time of data collection (21 days after infection) an interruption of necrosis was detected in the edited plants and new shoots developed without symptoms, while control plants showed a continuous progression of the disease (Figure 4b).

### Screening of exogenous DNA elimination in heat‐shock‐treated edited apple plants

**Figure 5:**
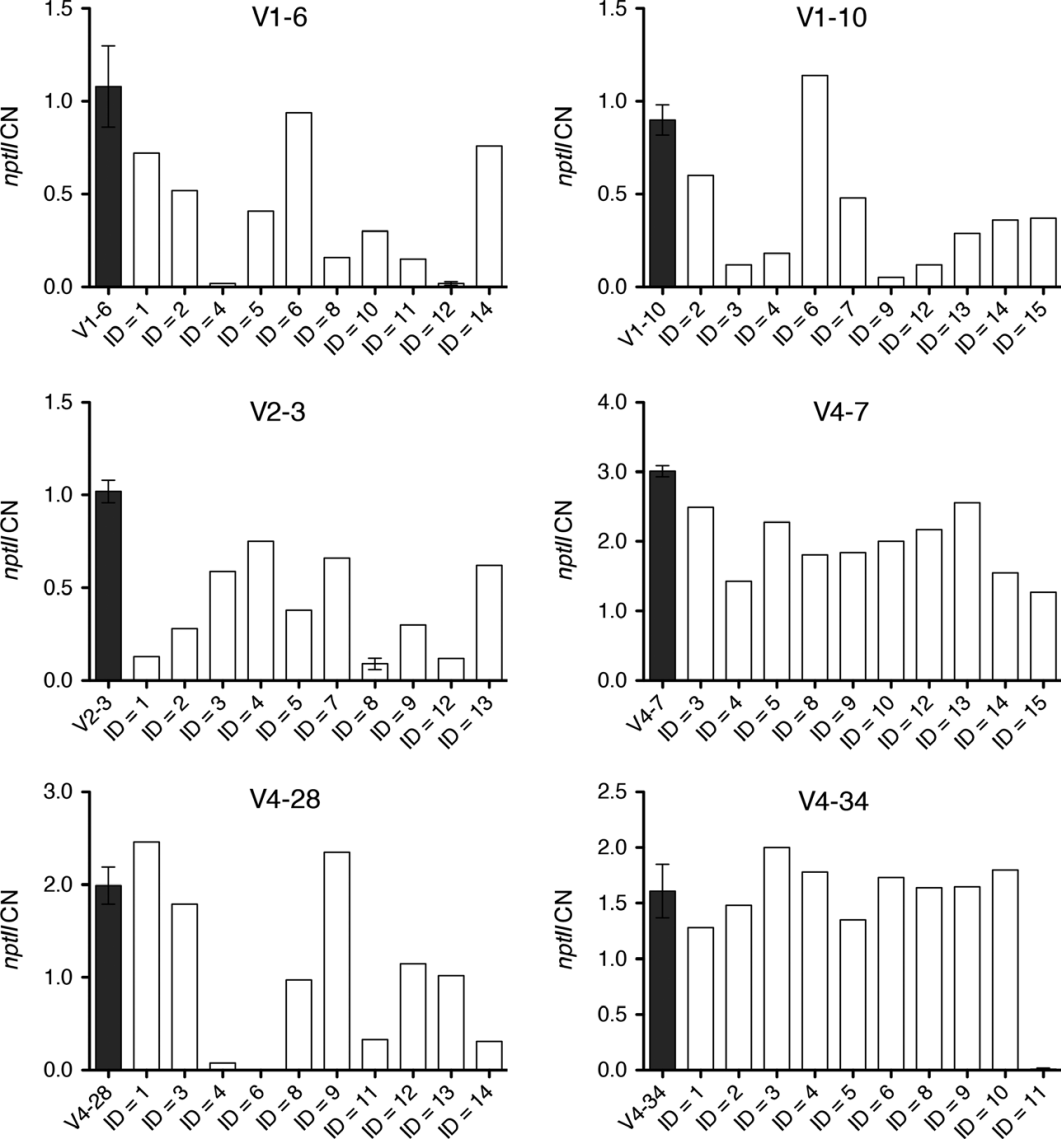
Summary of the *nptII* removal in heat‐shock‐treated *MdDIPM4*‐edited apple plants with reduced susceptibility to fire blight. *In vitro* biological replicates (*n* = from 6 to 10) of six candidate lines were heat‐treated, and the two central nodes of each plant were subsequently propagated. After 1 month, 10 new shoots regenerated from induced nodes were analysed by TaqMan real‐time PCR to quantify *nptII* copy number (CN). *NptII* CN of untreated plants (dark grey bars) and plants V1‐6.12, V2‐3.8 and V4‐34.11 is the mean ± SD of three biological replicates while the other plants were analysed in a single biological replicate. Primer sequences are listed in Table S1.

Apple lines V1‐6, V1‐10, V2‐3, V4‐7, V4‐28 and V4‐34, which showed a complete *MdDIPM4* knockout and a reduced susceptibility to fire blight disease, were subjected to heat‐shock treatments in order to activate the FLP/FRT recombination system for the removal of T‐DNA flanked by the two FRT sites (10.6 kb; Figure 1). To check the level of exogenous DNA elimination, the marker gene *nptII*, a crucial element of the T‐DNA cassette, was quantified in shoots (10 for each line) regenerated from central nodes of heat‐induced plantlets (Figure 5). On average, for each edited line, 1–2 shoots showed a high percentage of T‐DNA excision (91%–100%) except for lines V4‐7. Among 60 plants, seven showed more than 90% of T‐DNA removal, 21 exhibited between 50% and 90%, 21 between 10% and 50%, while in 11 plants a removal of less than 10% was observed. Some plants showing *nptII* CN equal or near to zero (V1‐6.12, V2‐3.8, V4‐34.11) were propagated for 2 months and subsequently re‐tested. The confirmation of the CN values demonstrated that in those plants the removal of the T‐DNA was stable (Figure 5).

### Validation of the T‐DNA excision in edited apple line V4‐28

**Figure 6:**
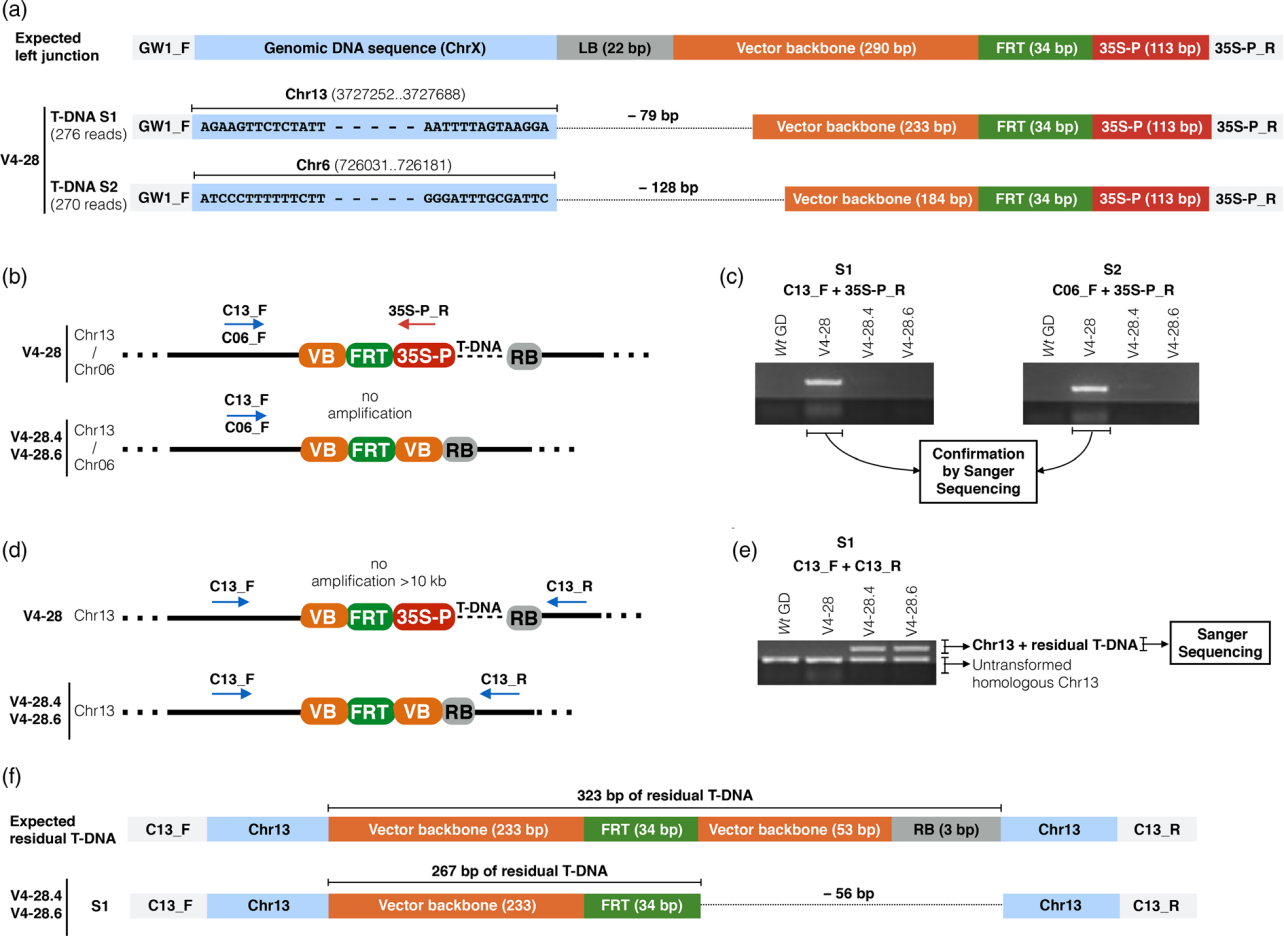
Identification of the T‐DNA insertion site and validation of the T‐DNA removal in the CRISPR/Cas9‐edited apple line V4‐28. (a) Visualization of the two T‐DNA insertion sites (S1‐2) identified by HTS on chromosomes 13 and 6, respectively. In both cases, compared to the expected sequence, the left end of T‐DNA was digested of 79 and 128 bps, respectively. (b) Schematic representation of the amplification expected with the combinations of primers C13_F + 35S‐P_R and C06_F + 35S‐P_R, in not‐treated V4‐28 plant and in heat‐shock induced shoots (V4‐28.4 and V4‐28.6). (c) Results of the PCR described in 6B. (d) Schematic representation of the amplification expected with primers C13, during the validation of the T‐DNA removal in S1. (e) Results of PCR described in 6D. (f) Visualization of the T‐DNA removal and corresponding residual T‐DNA in S1. GenomeWalker (GW); left border (LB); flippase recognition target site (FRT); *Cauliflower Mosaic Virus 35S* promoter (35S‐P); site (S); chromosome (Chr). Vector backbone (VB); right border (RB).

The apple line V4‐28 (CN = 1.99 ± 0.2; Figure 5) and relative heat‐induced clones V4‐28.4 and V4‐28.6, which showed a T‐DNA elimination, respectively, of 96% (CN = 0.08) and 100% (CN = 0.00), were selected to further demonstrate the T‐DNA excision by genomic sequencing. At first, the unheated clone V4‐28 was used to identify the predicted two T‐DNA insertion sites (Figure 6a) by a procedure combining an enrichment PCR with HTS as detailed in the experimental procedures section. The putative genomic DNA sequences derived from sequencing were blasted against the apple genome GDDH13 v1.1 (Daccord *et al*., 2017), and two sites with perfect sequence identities were found on chromosomes 13 (first T‐DNA insertion site, S1) and 6 (second T‐DNA insertion site, S2; Figure 6a). No gene was annotated at these two genomic sites. In addition, in S1 the T‐DNA left border (22 bps) and 57 bps of vector backbone were absent, and similarly, a 128 bps sequence of the T‐DNA left‐border region has been lost in S2 (Figure 6a). This truncation was probably due to the T‐DNA translocation mechanism during the *Agrobacterium*‐mediated plant transformation. The two identified T‐DNA insertion sites were confirmed by PCR with a specific forward primer annealing on the upstream genomic region (C13_F or C06_F) and with a reverse primer annealing on the promoter 35S (35S‐P_R; Figure 6b). Predictably, the amplification occurred only for the unheated plant V4‐28 and not for plants V4‐28.4 and V4‐28.6 which lost the 35S promoter after the heat‐shock induction (Figure 6c). The removal of exogenous DNA and resulting residual T‐DNA sequence were checked in apple clones V4‐28.4 and V4‐28.6 at the integration site 1 by means of a PCR amplifying the Chr13 region containing the T‐DNA insertion site (Figure 6d). As expected, for the plant V4‐28, characterized by the whole T‐DNA cassette, no amplification was obtained (the amplification fragment would have been >10 kb, a non‐amplifiable size with the conditions used in PCR). On the contrary, in plants V4‐28.4 and V4‐28.6 the amplification occurred (Figure 6e). In all plants tested, a band (206 bps; Table S3) was visible due to the amplification of the corresponding DNA fragment on the *wild‐type* copy of homologous chromosome 13. DNA bands corresponding to the fragment of interest (Chr13 + residual T‐DNA) were gel purified and Sanger sequenced (Figure 6e). The previously identified digestion pattern at the left end of the T‐DNA cassette (79 bps) was validated, and similarly, a 56 bps sequence truncation was identified at the right end (Figure 6f).

### Detection of the CRISPR/Cas9‐editing activity in off‐target genomic sites

**Figure 7:**
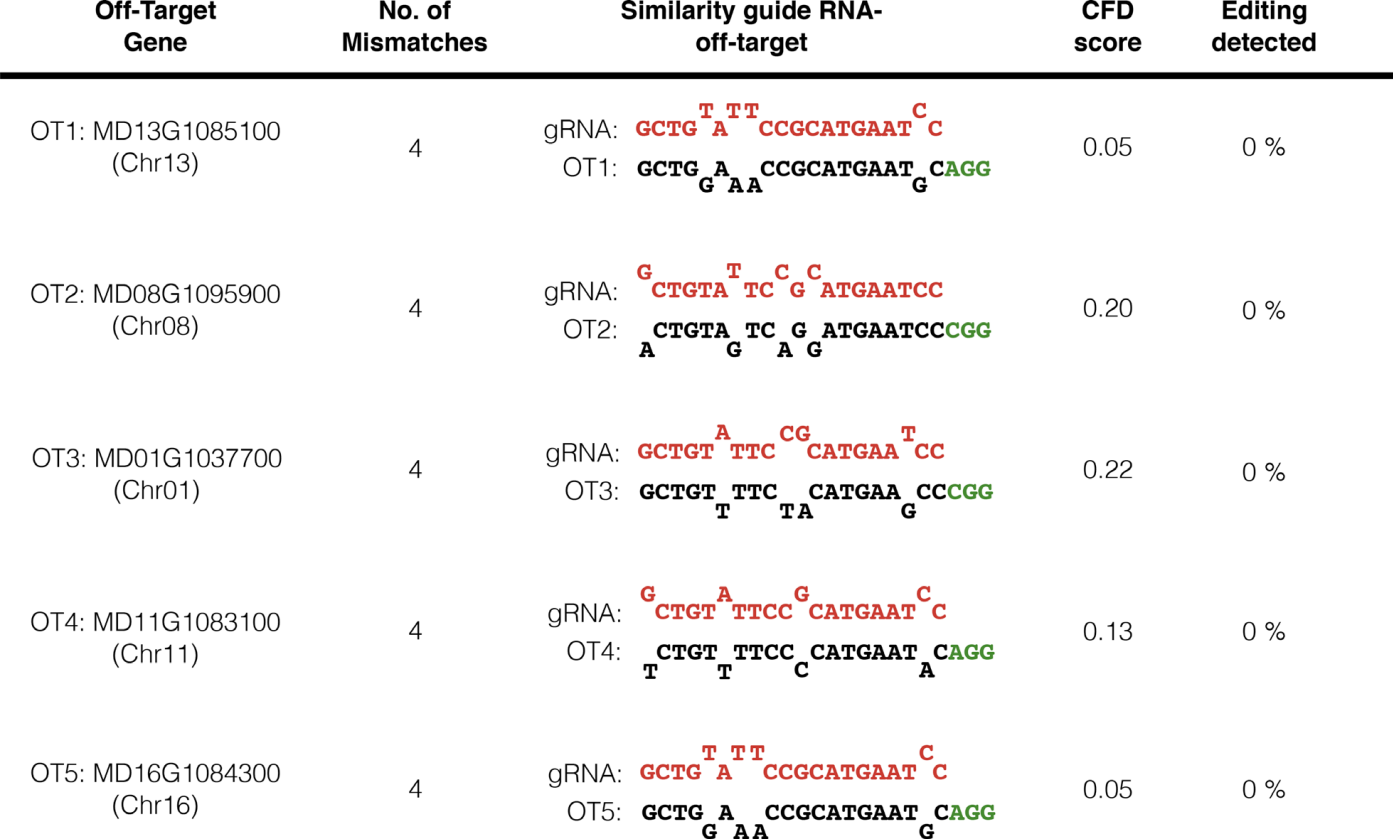
Summary of the CRISPR/Cas9 off‐targets analysis. The putative off‐target genomic sites of the guide RNA were predicted with the CRISPOR Software (http://crispor.tefor.net; Haeussler *et al*., 2016), using the apple genome assembly GDDH13 v1.1 (Daccord *et al*., 2017) as reference parameter. Five predicted off‐targets (OT1‐5) were selected according to the annealing features with the guide RNA and relative CFD scores. Genomic DNA fragments containing selected OT sites were amplified in six apple mutant lines and screened by HTS. No editing was detected in any of the tested lines. The percentage of editing is representative of all the analysed six lines. Sequences of the guide RNA, off‐target region and PAM site are shown, respectively, in red, black and green. Primer sequences are listed in Table S4.

In addition to the heat‐shock treatments, apple lines V1‐6, V1‐10, V2‐3, V4‐7, V4‐28 and V4‐34 were further investigated to screen the editing activity of the CRISPR/Cas9 machinery on potential off‐target (OT) genomic sites (Figure 7). The prediction of the OT regions revealed that our guide RNA was highly specific as no target was found with 1, 2 and 3 mismatches. However, 7 OT sites showing four mismatches with the guide RNA were predicted. Among them, five OTs (Figure 7) characterized by different Cutting Frequency Determination (CFD) scores were selected and screened by HTS. The obtained raw reads (ca. 6000/OT/plant) were processed and visualized to detect the OT editing. No mutation was identified in any of the 30 (six lines × five putative OT sites) tested samples (Figure 7).

## Discussion

Most of today's elite cultivars such as ‘Braeburn’, ‘Cripps Pink’, ‘Gala’, ‘Golden Delicious’, ‘Fuji’ and ‘Jonagold’ are susceptible to fire blight (Norelli *et al*., 2003). To manage this disease, the use of resistant cultivars commercially available (e.g. ‘Rewena’ and ‘Enterprise’; Kellerhals *et al*., 2014; Richter and Fischer, 1999) is currently not widespread, as the quality of their fruits does not fulfil consumers demand. As alternative, in the last decade genetic engineering has been used as a strategy to combat fire blight in apple by introducing resistance gene from crab apple genotypes into cultivars of interest. Broggini *et al*. (2014) transferred the *FB_MR5* gene, deriving from the wild apple accession *Malus* ×* robusta* 5, into the susceptible genotype ‘Gala’ *via A. tumefaciens* and observed a reduction in disease symptoms (on average 80%) in transgenic lines after inoculation with two different *E. amylovora* strains. Using the same gene, Kost *et al*. (2015) produced the first cisgenic ‘Gala’ apple plant showing a reduction in fire blight susceptibility between 50% and 80%. However, it is known that the pathogen can overcome this resistance by a non‐synonymous single nucleotide mutation in the *AvrRpt2EA* effector gene responsible for an amino acid exchange (C156S) in the protein (Broggini *et al*., 2014; Vogt *et al*., 2013). In general, inherited resistance has been shown to be potentially overcome within years through pathogen mutation, hence requiring constant production of apple plants with new resistance traits for long‐lasting disease management (Aldwinckle and Beer, 1997; Lespinasse and Aldwinckle, 2000; Mundt, 2014). The knockout of susceptibility genes, which are required for compatible plant–pathogen interaction and for successful infection, has recently been considered a promising alternative strategy to breeding resistant plants (Pavan *et al*., 2010; Zaidi *et al*., 2018), potentially leading to more durable plant protection compared to that based on resistance genes. Campa *et al*. (2018) silenced the susceptibility *HIPM* gene and obtained around 50% of reduction in fire blight symptoms. In our study, we knocked out *MdDIPM4* in two commercial apple cultivars to confirm the hypothesis that this gene is associated with fire blight susceptibility. A highly significant reduction in symptoms was observed in edited plants compared to control (Figure 4), highlighting the importance of *MdDIPM4* in the onset of the disease. Our data confirmed the preliminary results of Borejsza‐Wysocka *et al*. (2004) who used RNA interference to silence DIPM family with the difference that, while in all cases these authors cross‐silenced two or more *DIPM* genes, in our work the CRISPR/Cas9 system targeted a specific region of *MdDIPM4* with no homology to other members of the family, ensuring a gene‐specific knockout (Figure S1). Overall, the knockout of *MdDIPM4* resulted in a highly significant reduction in susceptibility, on average 50% in both cultivars. This is a valuable result in view of managing this disease in the future allowing to reduce chemical treatments for a sustainable agriculture. Even if the percentage of symptom reduction is slightly lower than those obtained by Broggini *et al*. (2014) and Kost *et al*. (2015) by exploiting resistance genes (almost 80% and 50%–80%, respectively), the approach we proposed may be more long‐term effective. Moreover, considering the work of Campa *et al*. (2018) a promising strategy would be the simultaneous knockout with CRISPR/Cas9 of the susceptibility genes *HIPM* and *DIPM4*, involved in different cellular processes.

Genome editing is a revolutionary technology in molecular biology, able to introduce mutations into a plant gene in a rapid and highly specific manner. Recently, the CRISPR/Cas9 system was successfully applied to gene functional studies and molecular breeding in both woody and non‐woody plants (Andersson *et al*., 2017, Gil‐Humanes *et al*., 2017, Jia and Wang, 2014; Liang *et al*., 2017; Malnoy *et al*., 2016; Nishitani *et al*., 2016; Pan *et al*., 2016; Ren *et al*., 2016; Ueta *et al*., 2017; Wang *et al*., 2018). Nevertheless, especially in apple, the potentiality of this editing system has to be further explored. Following the *A. tumefaciens*‐mediated transformation, many plant lines were obtained, almost all with the T‐DNA cassette integrated, indicating that the method used is efficient (Table 1). To estimate the editing performance, we sequenced the *MdDIPM4* target site by both Sanger sequencing and HTS (Figures 2 and S2). Our data demonstrated that HTS is a more effective, rapid and cost‐efficient tool compared to Sanger sequencing for characterizing the profile of the target gene in edited plants. Nishitani *et al*. (2016) sequenced by Sanger method an average of more than 40 clones/putatively edited plant with great expense in terms of time and costs, while Charrier *et al*. (2019) only sequenced a mean of four clones/plant, a narrow spectrum to detect all possible mutation variants. On the contrary, our methodology, based on a HTS, allowed the simultaneous visualization of thousands of virtual clones for a single plant for less than $40. By using our HTS‐based approach, new editing profiles were found for the lines V1‐6 and V4‐7 compared to previous data obtained with Sanger method (five clones/plant), supporting the need of HTS for genotyping CRISPR/Cas9‐edited plants.

Previous studies have shown that the editing efficiency and mutation types associated with CRISPR/Cas9 can vary widely, depending on the transformation method, plant species, target sequence, Cas9 promoter and guide RNA (Ma *et al*., 2016). In our work, the obtained editing efficiency and mutations (Figure 2) were not cultivar‐dependent, as no important variations were observed between the two cultivars used. The editing efficiency we obtained (around 70% for both cultivars) is definitively higher compared to those found by Malnoy *et al*. (2016) (3.3%) and Nishitani *et al*. (2016) (31.8%). In the first case, it could be due to the low editing efficiency of protein–RNA complexes introduced into plant cells by polyethylene glycol (PEG) compared to classical methods (Metje‐Sprink *et al*., 2019). In the second case, the use of a different promoter regulating the expression of the Cas9 protein or of a different guide RNA could be the cause. In addition, our data regarding the kinds of mutations were in agreement with the previous work of Malnoy *et al*. (2016), who tested the editing produced in *DIPM* genes by Cas9‐guide RNA delivered as ribonucleoproteins in protoplasts of the apple cultivar ‘Golden Delicious’. In fact, small deletions (−1, −2 and −3 nts) were mostly identified with only few cases of small insertions (+1, +2 and +3 nts). The types of mutations detected in our work were also in agreement with those reported in the study of Nishitani *et al*. (2016), who targeted the *PDS* gene of the apple rootstock ‘JM2’. On the contrary, in the work of Charrier *et al*. (2019), who edited *PDS* and *TFL1* genes of the apple cultivar ‘Gala’, a small insertion of 1 nt was the most abundant mutation in the two target sites of *PDS* gene. However, small deletions (−1, −2 and −4 nts) were mostly found in the two CRISPR/Cas9‐targeted regions of *TFL1* gene. According to our results, it can be supposed that in apple, the cell repair system for double‐strand break tends to preferentially produce deletions regardless of the CRISPR/Cas9 system variants (e.g. delivery methods, guide RNA, elements of the binary vectors). On the other hand, these variants are crucial to determine the efficiency of editing.

The CRISPR/Cas9 system has become a universal powerful tool for targeted gene manipulation. However, its application is associated with off‐targeting, that is the generation of unwanted mutations in off‐target genomic sites. In *Arabidopsis* (Zhang *et al*., 2018), barley (Lawrenson *et al*., 2015), *Brassica oleracea* (Lawrenson *et al*., 2015), rice (Tang *et al*., 2018), apple (Charrier *et al*., 2019) and pear (Charrier *et al*., 2019), the occasionally off‐targeting has been reported to be a potential issue when CRISPR/Cas9 was applied, especially if the guide RNA shared significant similarity (complete homology or 1 mismatch) with OT sequences. On the contrary, in apple (Charrier *et al*., 2019) and grape (Wang *et al*., 2018) no editing activity was found by the analysis of OT regions showing three or four mismatches with the guide RNAs. In our work, the guide RNA was highly specific as only OT sequences with four or more mismatches were predicted. Five OTs were selected and screened by HTS in six candidate apple lines. As hypothesized, no mutations were detected in any of the sample tested (Figure 7) confirming that off‐targeting tightly depends on the number of mismatches. These data suggest that a right selection of the target sequence, based on the genome information available for that particular plant species, is the first requirement to avoid the CRISPR/Cas9 off‐target activity.

Among applications of the CRISPR/Cas9 system, the transformation mediated by *A. tumefaciens* is an effective system for achieving targeted mutations. However, this method leads to the production of transgenic edited plants. In Europe, genome‐edited organisms are to be considered GMO and must be subject to GMO legislation even if free of exogenous DNA (according to European Court of Justice sentence, July 2018). Many other countries such as the USA, Argentina, Australia and Brazil have established that genome‐edited cultivars that do not contain foreign DNA will not be subject to additional regulatory oversight and risk assessment as required for GMO (Eriksson *et al*., 2019). The approach used by Charrier *et al*. (2019), based on *A. tumefaciens* transient transformation and on a high‐throughput screening of the T‐DNA‐free edited plants, may not be feasible for the editing of target sites that do not lead to a visual plant phenotype. These authors, in an experiment aimed at knocking out *PDS* gene in apple, had to regenerate 747 shoots to observe three albino events. Another strategy is based on a site‐specific excision mechanism to remove a region of DNA. Several systems exist, such as Cre/loxP, R/Rs or FLP/FRT, based on recombinase enzymes that recognize two directly repeated RRS and excise the region within leaving in the plant genome a single 34 bps RRS. In apple, the application of these systems for the removal of selection marker genes was successfully reported (Herzog *et al*., 2012; Kost *et al*., 2015; Righetti *et al*., 2014). Other systems may be used like the insect PiggyBac transposon, which do not leave exogenous scars in the plant genome after excision. However, PiggyBac transposon has been poorly exploited in plant, proving a good efficiency only in rice (Nishizawa‐Yokoi *et al*., 2014, 2015).

In our study, we focused on the removal of the T‐DNA cassette in those CRISPR/Cas9‐edited lines with reduced fire blight susceptibility by using the FLP/FRT system, inducible with a heat‐shock stimulus. The T‐DNA cassette removal in six selected lines treated with heat shock was preliminarily evaluated by estimating the CN of *nptII*, the selection marker gene placed inside the T‐DNA cassette (correlating its CN reduction with the removal of the cassette itself; Figure 5). Following the heat‐shock inductions, 10 regenerated plants/line were screened and the removal was shown to occur in all analysed lines with percentages that reached also 100% (Figure 5). These data, compared with those of previous studies (Herzog *et al*., 2012; Kost *et al*., 2015; Righetti *et al*., 2014), demonstrated that our method is efficient and rapid, as it does not require the treatment of thousands of leaf explants as well as callus regeneration which are laborious and time‐consuming. To further validate the T‐DNA excision and characterize the corresponding residual sequence after the FLP/FRT‐mediated recombination event, the apple line V4‐28 was investigated by sequencing for the identification of the predicted two T‐DNA insertion sites (Figure 6). Insertions were found on Chr13 and Chr6, respectively. Thus, the insertion on Chr13 was selected and sequenced in *nptII*‐free clones V4‐28.4 and V4‐28.6. Compared to the expected sequence, 135 bps of T‐DNA ends were trimmed away (79 bps on left side and 56 bps on right side) and a residual exogenous DNA sequence of 267 bps was confirmed. In cisgenic apples, similar trimming patterns were already reported (Kost *et al*., 2015; Würdig *et al*., 2015). These results show that occasional border region truncations occur when the T‐DNA is translocated into the plant genome and their effect must be considered during the construction of transformation vectors. The system we propose does not totally eliminate the whole T‐DNA sequence (in the case of line V4‐28, it leaves a scar of 267 bps in Chr 13 instead of the 402 bps predicted), and this aspect is still going to clash with strict legislations in many countries worldwide. However, successful deletion of a big region of T‐DNA (more than 97% of its length) containing the CRISPR‐Cas9 editing machinery may be promising for this important fruit crop where available tools for efficient genome engineering are still limited.

In conclusion, we have developed and applied a CRISPR/Cas9‐FLP/FRT gene editing system to produce edited apple cultivars with reduced fire blight susceptibility and carrying a minimal trace of exogenous DNA. Overall, our data confirm that *MdDIPM4* is involved in apple susceptibility to fire blight and that the inactivation of this single gene of the DIPM family is sufficient to significantly reduce disease symptoms. Moreover, T‐DNA removal allows to eliminate the CRISPR/Cas9 from the genome in view of protecting plants from any effect due to the presence of an exogenous endonuclease and, simultaneously, to repeat gene transfer rounds on the same plants using the same selection marker gene. This methodology could represent a promising alternative strategy to the classical breeding for transgene introgression, especially for those plant species (such as apple) which require long maturation and crossing times. Plants produced in this work could be further investigated to better understand how *MdDIPM4* is involved in the onset of fire blight disease.

## Experimental procedures

### Construction and mechanism of action of the p9‐C‐HS‐D4 binary vector

The 17 kb p9‐C‐HS‐D4 binary vector (abbreviation of p9‐Crispr/Cas9 heat‐shock *MdDIPM4*) (Figure 1) was designed by us and assembled by the ‘DNA Cloning Service e.K.’ (Hamburg, Germany). The T‐DNA cassette, flanked by the left and right borders (grey boxes; Figure 1), incorporated three distinct molecular systems, respectively, for antibiotic resistance, gene editing and T‐DNA excision. The antibiotic resistance system (red boxes; Figure 1) was characterized by the *Neomycin phosphotransferase II* gene (*nptII*), driven by the *Cauliflower Mosaic Virus 35S* promoter, which conferred kanamycin resistance to apple transformants during the antibiotic‐assisted selection following transformation (step 1; Figure 1). The gene editing system (blue boxes; Figure 1) was based on the *wild‐type CRISPR‐associated protein 9* gene from *Streptococcus pyogenes*, controlled by the *Arabidopsis thaliana Ubiquitin‐10* promoter, and the 20 bps guide RNA (5′‐GCTGTATTCCGCATGAATCC‐3′; Malnoy *et al*., 2016) for the target of exon 2 of *MdDIPM4* (yellow box, step 2; Figure 1, 2a and S2), driven by the *Arabidopsis thaliana U6* promoter. Finally, the T‐DNA excision system (green boxes; Figure 1) consisted of the FLP/FRT recombinase of *Saccharomyces cerevisiae* combined to the heat‐shock inducible promoter of the soya bean gene *Hsp17.5‐E* (Czarnecka *et al*., 1989). The system was designed with the *Flippase* gene under the control of the heat‐shock inducible promoter and the two *Flippase Recognition Target* sites next to the left and right borders in order to remove the entire T‐DNA cassette (leaving in the apple genome a predicted exogenous DNA sequence of 402 bps; step 3; Figure 1).

### Apple transformation and identification of transgenic plants

Competent cells of *Agrobacterium tumefaciens* strain EHA105 (Hood *et al*., 1993) were transformed by electroporation with the p9‐C‐HS‐D4 binary vector and used to transform plantlets of *Malus* × *domestica*, cultivars ‘Gala’ and ‘Golden Delicious’, as described by Joshi *et al*. (2011). Transformations were performed in duplicate (V1 and V3 for ‘Gala’; V2 and V4 for ‘Golden Delicious’; Table 1) using from 300 to 1000 leaf explants (Table 1). Regenerated plants (obtained after 6–7 months from co‐culture with *Agrobacterium*) were screened to detect the presence of the T‐DNA cassette (Table 1). For each plant, genomic DNA was extracted from 2 leaves using the Illustra™ Nucleon DNA Extraction Kit PHYTOPURE™ (GE Healthcare), quantified on the NanoDrop 8000 Spectrophotometer (Thermo Fisher Scientific), diluted to 50 ng/μL and used for PCRs using the thermocycle‐3000 (Biometra), the GoTaq^®^ Green Master Mix 2X (Promega, Fitchburg, MA) and primers Cas9, VirG and MdTOPO6 (0.4 μm) listed in Table S1.

### Detection of the *MdDIPM4* editing by HTS

The *MdDIPM4* CRISPR/Cas9‐targeted region of 57 transgenic apple lines (27 for ‘Gala’ and 30 for ‘Golden Delicious’) and *wild‐type* plants was massively screened by HTS (Figure 2a). The *MdDIPM4* region containing the target site was amplified with primers MdDIPM4(2) (0.4 μm; Table S1) and overhang Illumina adapters to generate the Illumina library amplicons and sequenced on an Illumina MiSeq (PE300) platform (MiSeq Control Software 2.0.5 and Real‐Time Analysis Software 1.16.18) as reported by Quail *et al*. (2012). The CRISPResso pipeline (http://crispresso.rocks/; Pinello *et al*., 2016) was used to process (with default parameters) the raw paired‐end reads, contained into ‘fastq’ files, and to visualize the mutations profiles in *MdDIPM4* target sequence (Figure 2a).

### Quantification of the *nptII* CN by TaqMan real‐time PCR

The quantification of the *nptII* CN was used to quantify T‐DNA insertion (Table S2) and subsequently to assess the removal of exogenous DNA (Figure 5) in selected candidate apple lines following heat‐shock inductions. The experimental procedure was conducted according to the TaqMan real‐time PCR method developed by Dalla Costa *et al*. (2019), and primers and probes for the endogenous gene *MdTOPO6* and for the marker gene *nptII* are listed in Table S1.

### Plants resistance test to *Erwinia amylovora*

Resistance to *E. amylovora* was determined according to the scissor inoculation method described by Desnoues *et al*. (2018). From 3 to 15 biological replicates for each plant line were inoculated with *E. amylovora* strain Ea273 (10^9 ^CFU/mL) in each of the three independent experiments performed. Only actively growing plants that showed a shoot length of at least 13.0 cm were considered for the experiments. Data collecting was performed according to Campa *et al*. (2018). Statistical analysis was performed using the Dell™ Statistica™ Software version 13.1, considering ‘Gala’ and ‘Golden Delicious’ data sets separately (Figure 4). As the three experiments showed the same trend, measures of each plant line were merged and analysed as a single experiment. Measures which differed more than ±1.5 interquartile ranges from the mean of the relative group were considered outliers (Figure 4). Nonparametric Kruskal–Wallis test was used to compare groups as data did not show a normal distribution. Subsequently, all groups were compared simultaneously by multiple comparisons of mean rank. Statistical analysis was performed with α = 0.05.

### Heat‐shock induction of the FLP/FRT recombination system

The induction of the FLP/FRT recombination system was based on Herzog *et al*. (2012) and Dalla Costa *et al*. (2016). From six to ten 2‐week‐old plants for each line were incubated three times at 42 °C for 6‐h with a 48‐h interval between consecutive incubations in a hybridization oven ‘hybridizer HB‐1000’ (UVP, Upland, CA). At the end of the heat‐shock inductions, leaves, the vegetative apex (in most of the cases necrotic) and first 1–2 basal internodes of each plant were cut and discarded. The two central nodes of the stem were collected and placed horizontally onto a fresh propagation medium to promote the regeneration of new shoots. After 1 month, the first two leaves of 10 regenerated shoots for line were collected for DNA extraction and *nptII* quantification (Figure 5), according to the method previously described. Apple clones V1‐6.12, V2‐3.8 and V4‐34.11 were micropropagated twice, and the new shoots were re‐tested for *nptII* CN.

### Identification of the T‐DNA genomic insertion site

Genomic DNA (1 μg extracted from one unheated biological replicate of the plant line V4‐28) was subjected to three low‐intensity sonication cycles of 30‐s with 90‐s interval on a Bioruptor^®^ NGS (Diagenode). Sonicated DNA was purified according to 1.8× AMPure XP Beads protocol (Agencourt) and subsequently checked on a D1000 ScreenTape (Agilent) to confirm the DNA fragmentation between 200 and 1000 bps. DNA fragments ends were repaired with the NEBNext^®^ End Repair Module E6050S (New England Biolabs), following the manufacture's instructions, and the resulting DNA solution was again purified and checked, as mentioned above. The purified genomic DNA fragments were ligated to GenomeWalker adaptors, according to the procedure of the Universal GenomeWalker™ 2.0 kit (Takara Bio), and subsequently used in a selective PCR with primers GW1_F and 35S‐P_R (0.4 μm; Table S3) to amplify those fragments containing the junction between the genomic DNA and the left end of T‐DNA (Figure 6a). The PCR product was purified with 0.8× AMPure XP Beads and checked on a D1000 ScreenTape to validate the removal of DNA fragments smaller than 200 bps. Thus, the amplicon library product was sequenced by MiSeq Illumina platform, as previously mentioned. The obtained reads were visualized with the Unipro UGENE Software v1.31.1 (Okonechnikov *et al*., 2012) and checked to identify those containing the 35S‐P_R primer sequence. From the selected reads, all the recognizable vector sequence was removed and the remaining flanking unknown sequence (putative genomic DNA) was blasted against the apple genome assembly GDDH13 v1.1 (Daccord *et al*., 2017) in order to identify putative T‐DNA genomic insertion sites (Figure 6a). Following the identification, one genome‐specific forward primer/insertion site (C13_F and C06_F; Table S3) was designed to anneal a genomic DNA sequence ca. 100 bps upstream the identified T‐DNA insertion site. Thus, the genomic DNA of the unheated plant V4‐28, of the heat‐induced clones V4‐28.4 and V4‐28.6 and of *wild‐type* control was amplified by PCR with primers C13_F + 35S‐P_R or C06_F + 35S‐P_R (0.4 μm; Figure 6b) and PCR products were checked by 1% agarose gel electrophoresis. The obtained DNA bands (Figure 6c) were gel purified with the NucleoSpin^®^ Gel and PCR Clean‐up kit (Macherey‐Nagel) and directly sequenced by the method of Sanger, as mentioned before. The obtained reads were visualized with UGENE Software to confirm the previous HTS results.

### Validation of the T‐DNA removal

The validation of the T‐DNA removal was conducted in plants V4‐28.4 and V4‐28.6 which proved to be free from *nptII*. A genome‐specific reverse primer/insertion site (C13_R; Table S3) was designed to anneal a genomic DNA sequence ca. 100 bps downstream the T‐DNA insertion site. Thus, the genomic DNA of plants in object, relative V4‐28 and *wild‐type* controls was amplified by PCR using the couple of primers C13_F + C13_R (0.4 μm; Figure 6d) and the corresponding PCR products were electrophoretically checked, gel purified and Sanger sequenced (Figure 6e), as described in the previous paragraph.

### Off‐target analysis

Genomic DNA was extracted, as previously reported, from one biological replicate of each plant line analysed and the corresponding *wild‐type* plant. DNA fragments containing the off‐target sites (Figure 7) were amplified by PCR, with primers OT1‐5 (0.4 μm) listed in Table S4, and the amplicon libraries were sequenced by MiSeq Illumina Platform according to the *MdDIPM4* on‐target analysis. Raw paired‐end reads were analysed with CRISPResso using default parameters.

## Accession numbers

Genes of *Malus* × *domestica* can be found in the GDR Database under accession numbers: MDP0000948404, MDP0000229861, MD01G1037700, MD08G1095900, MD11G1083100, MD13G1085100 and MD16G1084300. Genes of *Arabidopsis thaliana* can be found in the TAIR Database under accession numbers: AT4G05320 *and* AT3G13855. Additional genes can be found in the EMBL/ENA Database under accession numbers: AAB59340.1, M28070.1 *and* X62885.1. The *SpCas9* nucleotide sequence is available at www.dna-cloning.com.

## Conflict of interest

No conflict of financial interest declared.

## Author contributions

V.P. designed the experiments, conducted the experiments and wrote the paper. L.D.C. contributed to designing the binary vector, the experiments and revised the paper. S.P. contributed to designing the experiments, performing the procedure for validating the T‐DNA removal and revised the paper. M.P. contributed to performing the sequencing analysis. M.M. designed the project, contributed to designing the experiments and revised the paper. All authors read and approved the paper.

## Supporting information


**Figure S1** Alignment of *MdDIPM* genes fragment corresponding to the target site of the CRISPR/Cas9 machinery.
**Figure S2** CRISPR/Cas9‐editing in *MdDIPM4* gene identified by Sanger Sequencing.
**Figure S3** Experimental workflow for the *Agrobacterium tumefaciens*‐mediated production of CRISPR/Cas9‐edited apple plants with reduce susceptibility to fire blight and ‘free’ from exogenous DNA.

**Table S1** Sequences of primers and probes used for the PCR‐based screening of apple transformants, the detection of CRISPR/Cas9 on‐target mutations and the quantification of *NPTII* copy number by Taqman real‐time PCRs.
**Table S2** Quantification by Taqman real‐time PCR of *nptII* copy number in CRISPR/Cas9‐edited apple lines.
**Table S3** Sequences of primers used in the identification of the T‐DNA genomic insertion sites and validation of the T‐DNA removal.
**Table S4** Sequences of primers used for the off‐target analysis.

**Data S1** Supporting Experimental Methods.
