# Supplementary material for: Reduced fire blight susceptibility in apple cultivars using a high‐efficiency CRISPR/Cas9‐FLP/FRT‐based gene editing system
Source: Plant Biotechnol J. 2019 Oct 3;18(3):845–58. doi: 10.1111/pbi.13253 (PMC7004915; doi:10.1111/pbi.13253)
Supplement: Supplementary file 1 — Figure S1 Alignment of MdDIPM genes fragment corresponding to the target site of the CRISPR/Cas9 machinery. Figure S2 CRISPR/Cas9‐editing in MdDIPM4 gene identified by Sanger Sequencing. Figure S3 Experimental workflow for the Agrobacterium tumefaciens‐mediated production of CRISPR/Cas9‐edited apple plants with reduce susceptibility to fire blight and ‘free’ from exogenous DNA. [file PBI-18-845-s002.docx]

**SUPPORTING FIGURES**

**Figure S1. Alignment of *MdDIPM* genes fragment corresponding to the target site of the CRISPR/Cas9 machinery.** Related to the section “Generation of edited transgenic apple lines” (Results). In the *MdDIPM4* genomic sequence, the PAM and the CRISPR/Cas9-targeted region are shown in green and blue, respectively. Asterisks indicate common bases between all the four genes.

**Figure S2.** **CRISPR/Cas9-editing in *MdDIPM4* gene identified by Sanger Sequencing.** Related to the section “Detection of the *MdDIPM4* editing by Sanger Sequencing” (Experimental procedures in Supporting Information). A total of 5 colonies for each transgenic and *wild-type* plant tested were screened in the analysis. In addition to *wild-type* plants, the *MdDIPM4* genomic sequence of the apple genome assembly GDDH13 v1.1 (Daccord et al., 2017) was used as control. The *MdDIPM4* target sequence is colored in blue. Within the aligned sequences, deletions and insertions are represented respectively with traits and black bold letters. Mutations, related number of colonies and plant ID are reported on the right. Guide RNA (gRNA); Protospacer Adjacent Motif (PAM). Primers sequences are listed in Table S1.

**Figure S3.** **Experimental workflow for the *Agrobacterium tumefaciens*-mediated production of CRISPR/Cas9-edited apple plants with reduce susceptibility to fire blight and ‘free’ from exogenous DNA.** Related to the section “Experimental workflow, plant material and growth conditions” (Experimental procedures in Supporting Information). The procedure requires almost 1.5 years to be completed. The five main steps are indicated with numbers 1 to 5 on the left.
