# Supplementary material for: Reduced fire blight susceptibility in apple cultivars using a high‐efficiency CRISPR/Cas9‐FLP/FRT‐based gene editing system
Source: Plant Biotechnol J. 2019 Oct 3;18(3):845–58. doi: 10.1111/pbi.13253 (PMC7004915; doi:10.1111/pbi.13253)
Supplement: Supplementary file 2 — Table S1 Sequences of primers and probes used for the PCR‐based screening of apple transformants, the detection of CRISPR/Cas9 on‐target mutations and the quantification of NPTII copy number by Taqman real‐time PCRs. Table S2 Quantification by Taqman real‐time PCR of nptII copy number in CRISPR/Cas9‐edited apple lines. Table S3 Sequences of primers used in the identification of the T‐DNA genomic insertion sites and validation of the T‐DNA removal. Table S4 Sequences of primers used for the off‐target analysis. [file PBI-18-845-s003.docx]

**SUPPORTING TABLES**

| **Primer and probe name** | **Sequence (5’-3’)** | **Amplicon length (bps)** | **Annealing temperature (°C)** |
| --- | --- | --- | --- |
| Cas9 | F: AGATCCTCACTTTTAGAATCCC  R: TGTCCTTGATAATCTTCAGGAG | 486 | 56 |
| VirG | F: GCCGGGGCGAGACCATAGG†  R: CGCACGCGCAAGGCAACC† | 605 | 56 |
| MdTOPO6 | F: TGTGGAAGGAGATCAAAGCGCA‡  R: CGCGTTGCTTCTTTGCTGCA‡ | 196 | 58 |
| MdTOPO6_probe | FAM-5′-ACATGCCAACAGGAACAATCACA-3′-TAMRA‡ |  |  |
| MdDIPM4(1) | F: CAATGTGGTGAGGCTGAGTG  R: GCTCGGTATCCTCCCAATCT | 598 | 61 |
| MdDIPM4(2) | F: GTGTTCAGTTTGGGGCACAT  R: GGAGGTTCTAACGGGGAGAG | 399 | 61 |
| NPTII | F: CTTGCCGAATATCATGGTGGAA§  R:GGTAGCCAACGCTATGTCCTGA§ | 100 | 58 |
| NPTII_probe | FAM-5′-TTCTGGATTCATCGACTGTGGC-3′-TAMRA§ |  |  |

† (Herzog et al., 2012)

‡ (Dalla Costa et al., 2019)

§ (Dalla Costa et al., 2009)

**Table S1. Sequences of primers and probes used for the PCR-based screening of apple transformants, the detection of CRISPR/Cas9 on-target mutations and the quantification of *NPTII* copy number by Taqman real-time PCRs.** Related to Table 1, Figure 2, Figure S2, Figure 5 and Table S2.

| **Gala** | |  | **Golden Delicious** | |
| --- | --- | --- | --- | --- |
| **Apple lines tested** | ***NptII* CN** |  | **Apple lines tested** | ***NptII* CN** |
| V1-2  V1-4  V1-5  V1-6  V1-7  V1-8  V1-10  V1-11  V1-12  V1-13  V1-14  V1-15  V1-16  V1-17  V1-18  V1-20  V1-21  V1-22  V1-23  V1-24  V3-1  V3-2  V3-3  V3-5  V3-6  V3-9  V3-10 | 0.8  1.1  0.7  1.4  1.8  0.2  0.9  0.9  1  2.3  0.6  1.2  1.5  0.8  0.9  2.1  1.3  0.9  0.7  1.3  2  0.8  0.7  2.1  1  4.5  0.8 | | V2-1  V2-3  V4-1  V4-2  V4-3  V4-4  V4-5  V4-7  V4-12  V4-13  V4-14  V4-15  V4-17  V4-19  V4-20  V4-21  V4-22  V4-24  V4-25  V4-26  V4-27  V4-28  V4-29  V4-30  V4-31  V4-32  V4-33  V4-34  V4-35  V4-36 | 3.4  1.1  4.9  1  0.4  1.8  0.6  3  1.5  0.8  2.8  0.8  1.5  2.1  0.3  1.1  2.7  0.8  1.6  1.5  0.8  2  0.7  1  2.4  0.7  1.1  1.7  0.9  0.9 |

**Table S2. Quantification by Taqman real-time PCR of *nptII* copy number in CRISPR/Cas9-edited apple lines.** Related to the section “Quantification of the *nptII* copy number by Taqman real-time PCR” (Experimental procedures). The *nptII* copy number, quantified in 1 biological replicate for each plant line analyzed, was used for the selection of candidate apple lines investigated in further analysis of plants resistance test to *E. amylovora* and heat-shock inductions. Copy number (CN). Primers sequences are listed in Table S1.

| **Primer name** | **Sequence (5’-3’)** | **Amplicon length (bps)** | **Annealing temperature (°C)** |
| --- | --- | --- | --- |
| GW1  35S-P  C13 | F: GTAATACGACTCACTATAGGGC†  R: GCTGGGCAATGGAATCCGAG  F: AGGTGGATTTTGTGGAAGGGA | 400-700  206 | 59  59 |
|  | R: AATAGCCACCGGATGAAGGG |  |  |
| C06 | F: CTGGGATTTGCGTTTCGAGT |  |  |

† (Universal GenomeWalker^TM^ 2.0 kit, Takara Bio)

**Table S3. Sequences of primers used in the identification of the T-DNA genomic insertion sites and validation of the T-DNA removal.** Related to Figure 6.

| **Primer name** | **Sequence (5’-3’)** | **Amplicon length (bps)** | **Annealing temperature (°C)** |
| --- | --- | --- | --- |
| OT1 | F: CGAACACGAGGGCTTTTCAT  R: AGATCGCACCACTTCTTCCA | 189 | 58 |
| OT2 | F: CAGGTAGTCGCCGGGATTTT  R: TTTCTATCTCCGGGGCCAGA | 89 | 60 |
| OT3 | F: TGATGTCTACAATGTGTACTTTGCT  R: GAGGCTATCCAGACCCAACG | 107 | 60 |
| OT4 | F: TCTGTAACAGCAATTTGGGAAAA | 191 | 58 |
| OT5 | R: ATCCAGGTCGTACTTTGTACC  F: TGCTTGTGCATTTCCTCTCG | 146 | 60 |
|  | R: CGAACTCGAGGGCTGTTCAT |  |  |

**Table S4. Sequences of primers used for the off-target analysis.** Related to Figure 7.
