# Supplementary material for: Reduced fire blight susceptibility in apple cultivars using a high‐efficiency CRISPR/Cas9‐FLP/FRT‐based gene editing system
Source: Plant Biotechnol J. 2019 Oct 3;18(3):845–58. doi: 10.1111/pbi.13253 (PMC7004915; doi:10.1111/pbi.13253)
Supplement: Supplementary file 3 — Data S1 Supporting Experimental Methods. [file PBI-18-845-s001.docx]

**SUPPORTING EXPERIMENTAL METHODS**

**Experimental workflow, plant material and growth conditions**

The experimental workflow was designed and conducted as described in Figure S3. For blocks 1, 2, 3, and 5 (respectively apple transformation, detection of CRISPR/Cas9-induced mutations, quantification of exogenous DNA, excision of exogenous DNA and off-target analysis - Figure S3), *in vitro* shoots of *M. x domestica* cultivars ‘Gala’ and ‘Golden Delicious’ were used. Baby jars containing apple plantlets in a shoot propagation medium (Pessina et al., 2016) were maintained in a growth chamber at 24 ± 1°C with a 16/8-h light/dark period (100 mmol/m2/s). For block 4 (*in vivo* plant resistance test to *E. amylovora* - Figure S3), soil-acclimatized apple plants of the same cultivars were used. To promote rooting, 2-week-old *in vitro* apple shoots were transferred from the shoot propagation medium to a Murashige and Skoog medium supplemented with indole‐3‐butyric acid (Pessina et al., 2016), maintained in a growth chamber in the dark for 2 weeks and subsequently under 16/8-h light/dark conditions, as described above, until complete root formation. Rooted plants were acclimatized in soil (‘Terriccio Vegetal Radic’ - TerComposti S.p.a., Brescia, Italy) and, by progressively reducing humidity for 3 weeks. Well-acclimatized plants were maintained at greenhouse conditions (24 ± 1°C, 16/8-h light/dark period, relative humidity of 70% ± 5%).

**Detection of the *MdDIPM4* editing by Sanger Sequencing**

The *MdDIPM4* genomic sequence containing the target site was screened in 10 transgenic apple lines and respective *wild-type* plants by Sanger Sequencing (Figure S2). DNA was extracted from leaves and amplified with primers MdDIPM4(1) (0.4 µM) (Table S1), as described in the previous paragraph. PCR products were purified with Nucleospin® Gel and PCR Clean-up Kit (Macherey-Nagel GmbH & Co.), quantified on the 2200 TapeStation System (Ag-lent Technologies) and cloned into *Escherichia coli* JM109 competent cells using a pGEM^®^-T Easy Vector (Promega), as in manufacturer’s instructions. Five positive colonies were selected for each plant line and corresponding PCR products were sequenced according to the method described by Sanger et al. (1977). Reactions were carried out on the ABI 3730xl DNA Analyzers (Applied Biosistems) following manufacturer’s instructions. Produced ‘SEQ’ files containing raw single-end reads generated by sequencing were processed and visualized with Unipro UGENE Software v1.31.1 (Okonechnikov et al., 2012), using default parameters for the quality of reads, to detect NHEJ mutations (Figure S2).

**References**

1. Dalla Costa, L., Vaccari, I., Mandolini, M. and Martinelli, L. (2009) Elaboration of a reliable strategy based on real-time PCR to characterize genetically modified plantlets and to evaluate the efficiency of a marker gene removal in grape (*Vitis spp.*). *J. Agric. Food Chem.* **57**, 2668–2677.
2. Pessina, S., Angeli, D., Martens, S., Visser, R.G.F., Bai, Y., Salamini, F., Velasco, R. et al. (2016) The knock-down of the expression of MdMLO19 reduces susceptibility to powdery mildew (Podosphaera leucotricha) in apple (Malus domestica). *Plant Biotechnol. J.* **14**, 2033–2044.
3. Sanger, F., Nicklen, S. and Coulson, A.R. (1977) DNA sequencing with chain terminating inhibitors. *Proc. Natl Acad. Sci. USA*, **74**, 5463–5467.
